# Supplementary material for: Identification of NHLRC1 as a Novel AKT Activator from a Lung Cancer Epigenome-Wide Association Study (EWAS)
Source: Int J Mol Sci. 2022 Sep 14;23(18):10699. doi: 10.3390/ijms231810699 (PMC9505874; doi:10.3390/ijms231810699)
Supplement: Supplementary file 1 [file ijms-23-10699-s001.zip › ijms-1896978-supplementary.pdf]

Supplementary Materials

Identification of *NHLRC1* as a novel AKT activator from a lung cancer epigenome-wide association study (EWAS)

1. Supplementary Tables

Table S1. Lung Tumor versus Adjacent Normal Replication Set II

**Lung Tumor versus Adjacent Normal Tissue (Replication Set II)**	
Total lung tumour-adjacent normal lung sample pairs (n)	24
Adenocarcinoma (n)	11 (pairs)
Squamous cell carcinoma (n)	13 (pairs)
Mean age at diagnosis (years)	63 (range: 49-81)
Men (n)	6
Female (n)	18
Current smokers (n)	6
Former smokers (n)	15
Neversmoker	2
Unknown smoking status (n)	1
Mean pack years (py)	56
Number of samples considered in the analysis (n)	48

Table S2. Primer Sequences, capital is the target, minuscule is tag sequence, Tm: melting temperature in °Celsius.

Name/Target	Primer Sequence 5'->3'	Tm [°C]
**MassARRAY (DNA methylation)**		
NHLRC1 F	aggaagagagATTGATTTAATTATGAGTATGATAGAAGAT	56
NHLRC1 R	cagtaatacgactcactatagggagaaggctACTCCCTATCACCCAACCTATAAAC	56
pCG-free_prom1.3-1 F	aggaagagagGATTAAAAGGAATTTTTGTAGGGTTT	58
pCG-free_prom1.3-1 R	cagtaatacgactcactatagggagaaggctAAATTACTCCCTATCACCCAACCTA	58
pCG-free_prom1.3-2 F	aggaagagagGTTTATAGGTTGGGTGATAGGGAGT	58
pCG-free_prom1.3-2 R	cagtaatacgactcactatagggagaaggctAATACCACTTTTCCTACACTACCCC	58
**Gene Expression (qPCR)**		
HPRT1_F	TGACACTGGCAAAACAATGCA	60
HPRT1_R	GGTCCTTTTCACCAGCAAGCT	60
GAPDH F	TGGACCTGACCTGCCGTCTA	60
GAPDH R	CCCTGTTGCTGTAGCCAAATTC	60
TRAILexpF	TTCACAGTGCTCCTGCAGTC	60
TRAILexpR	GCCACTTTTGGAGTACTTGTCC	60
CyclinG2exp1F	GGGGGTTGTTTTGATGAAAGT	60
CyclinG2exp1R	GATCACTGGGAGGAGAGCTG	60
NHLRC1 (transgen) F	TTGAGAAGTTTGGCCACCGG	60
NHLRC1 (transgen) R	CAGAATGGGCACTCGAGGG	60
NHLRC1 (endogenous) F	GCTCAGGTGGTCCTTCCAC	60
NHLRC1 (endogenous) R	TGGTAATGCACACTTGTGGTC	60
cGFP F	TCCTCCTTGAAATCGGTGCC	60
cGFP R	CGATCACATGAAGCAGCACG	60
**Cloning (overexpression)**		
pCRII-cGFP_NHLRC1_F	gttaaACCGGTATGGCGGCCGAAGCCT	58
pCRII-cGFP_NHLRC1_R	gttaaGGATCCTCACCCCCAGTCAACTTTATAGAC	58
**Site-directed mutagenesis**		
pCRII-cGFP-NHLRC1_C26Smut F	CTTCCAAAGCACACCTTAGACTCGAGCAGGCTGATCT	58
pCRII-cGFP-NHLRC1_C26Smut R	AGATCAGCCTGCTCGAGTCTAAGGTGTGCTTTGAGAAG	58
**Cloning (luciferase assay)**		
NHLRC1_clon_luc_enh_1_F	gttaaCCTAGGAGTGTGTTCGTGTTTTCCGG	61
NHLRC1_clon_luc_prom_3_R	gttaaAAGCTTTCACGGTCACAGTCATGGTC	61
**Sequencing**		
pCRII-cGFP_SEQ_F1	AGTCATCGCTATTACCATGG	58
pCRII-cGFP_SEQ_F2	TAACTAGAGAACCCACTGC	58
pCRII-cGFP_SEQ_F3	CAGATCAAGCTTGTCATTGG	58
pCRII-cGFP_SEQ_R	GCAACTAGAAGGCACAGT	58
pCpG-Lucia_seq	TCACAGTGCCACTTTTCCTG	58
**Pyrosequencing**		
NHLRC1_F	GGATAATAGGATTGTTTTTTGTTATTTAGT	
NHLRC1_R_biotin	ACCTCCCTTAAAATTTATCTTCTCT	
NHLRC1_S	GGTGTTTAGTAGTGTTTTTAAT	-

LINE1_F	TAGGGAGTGTTAGATAGTGG	
LINE1_R	AACTCCCTAACCCCTTAC	
LINE1_S	AACTCCCTAACCCCTTAC	-

2. Supplementary Figures

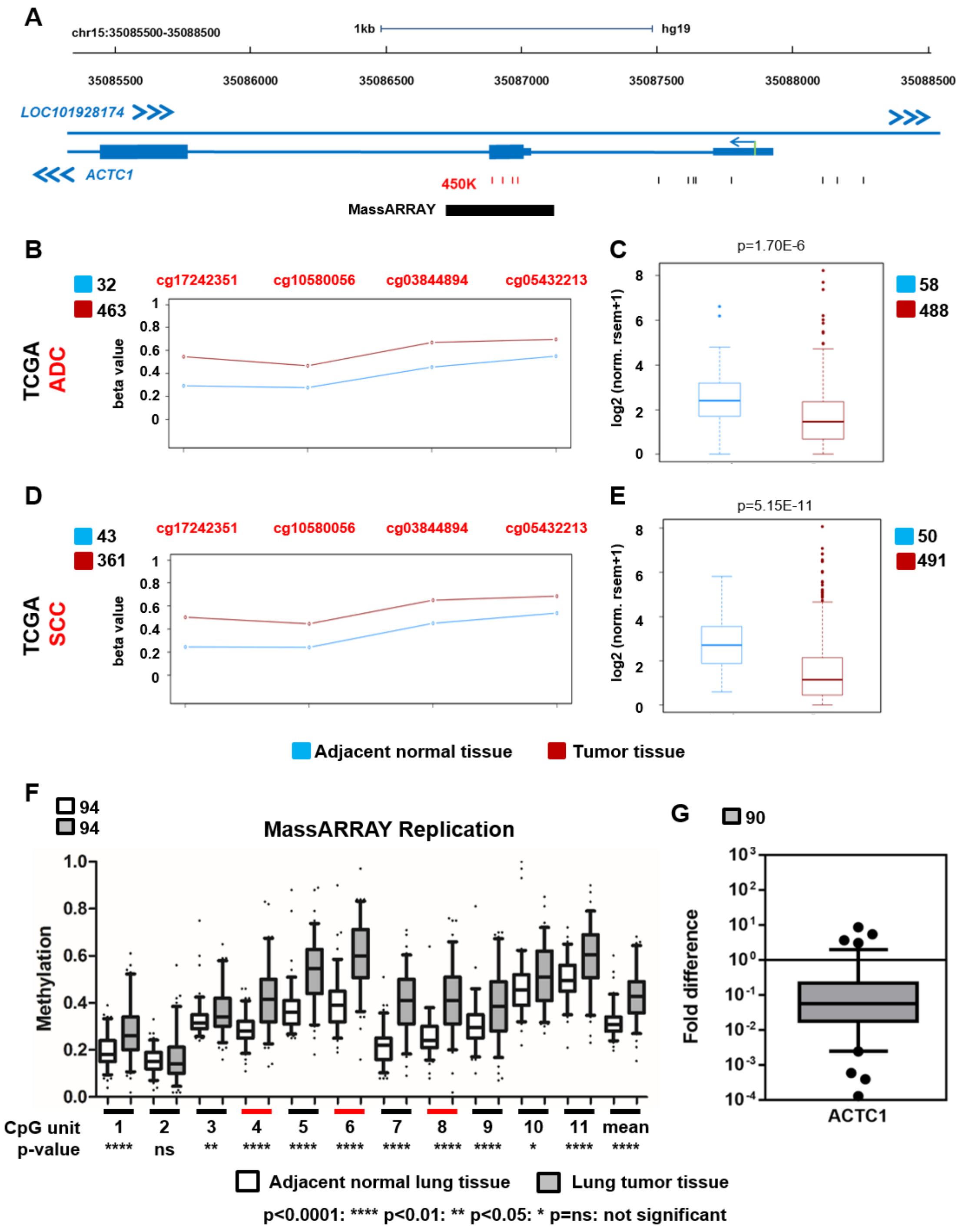

Figure S1. ***ACTC1 DMR M*ethylation and mRNA Expression in Lung Tumors. A:** Genomic location of the actin alpha cardiac muscle 1 (*ACTC1*) gene. The 399bp region tested by MassARRAY and the 450K CpG probe positions are indicated. Red ticks depict differentially

methylated 450K probes. **B, C:** *In silico* 450K TCGA data for lung adenocarcinoma (ADC; red) versus normal lung tissue (blue) depicting the DMR (B) and RNAseq data (C) for *ACTC1*. **D, E:** 450K TCGA data for lung squamous cell carcinoma (SCC; red) versus normal lung tissue (blue) depicting the DMR (D) and RNAseq data (E) for *ACTC1*. **F:** MassARRAY based replication in 94 paired lung tumor versus 94 adjacent normal lung samples to determine DNA methylation of all CpGs in a 399bp region outlined as CpG units. Red lines indicate those CpG units containing the CpGs which were originally identified as differentially methylated in EPIC and TCGA. Two tailed t-test p-values. **G:** *ACTC1* mRNA expression analysis by real time PCR in 90 samples of the lung tumor versus adjacent normal replication set. **F, G:** Whiskers indicate 5-95 percentile. norm.: normalized, rsem: RNA-Seq by Expectation Maximization. **B-E:** Images were produced using TCGA Wanderer platform and modified

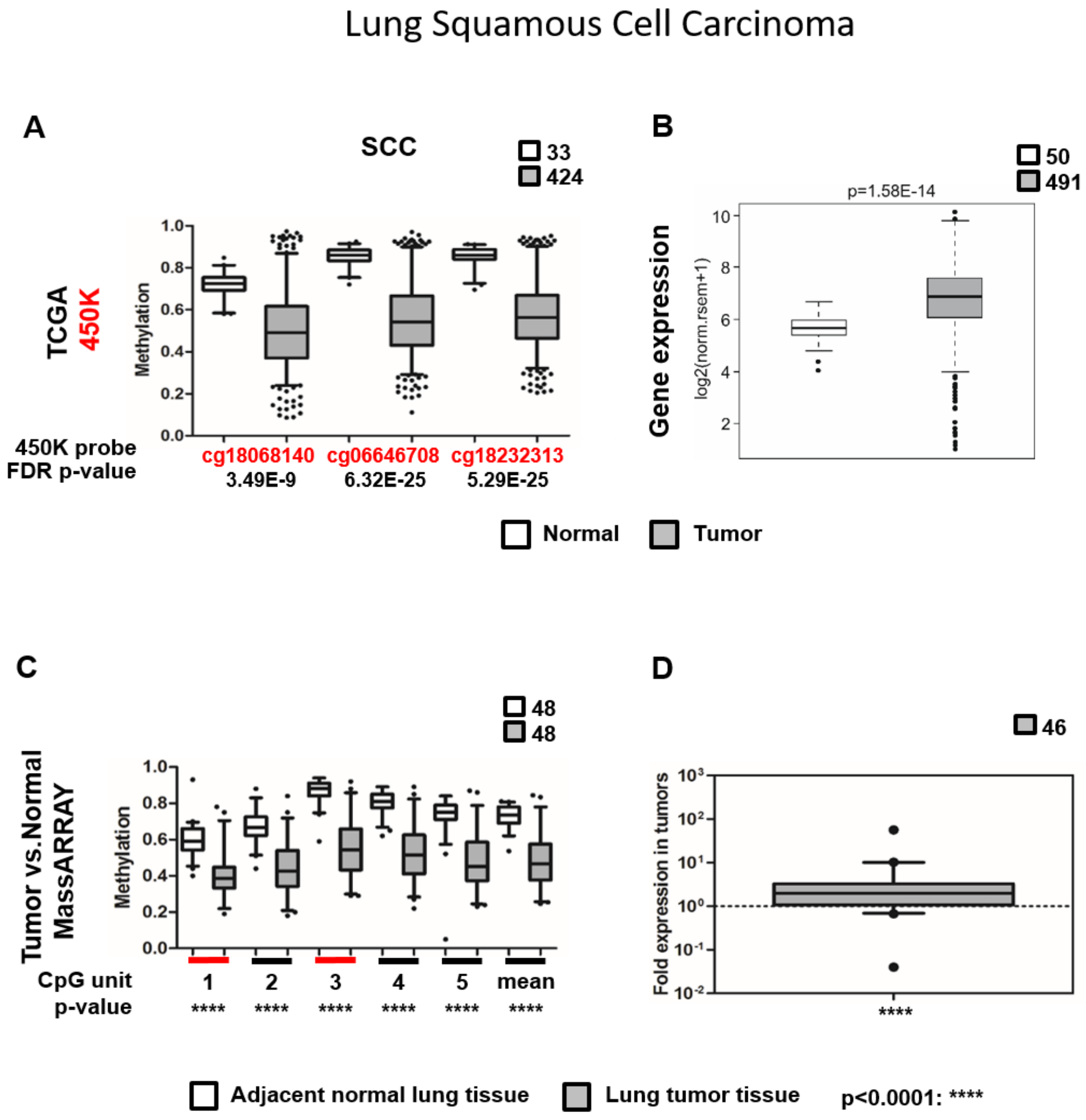

Figure S2. ***NHLRC1* DNA Methylation and mRNA Expression in Lung Squamous Cell Carcinomas. A:** *In silico* TCGA tumor versus normal 450K data analysis for SCC for the candidate and 2 adjacent CpGs in the NHLRC1 DMR. False discovery rate (FDR) corrected p-values are displayed. **B:** NHLRC1 gene expression of TCGA tumor versus normal data. **C.**

MassARRAY in 94 paired lung tumor versus 94 adjacent normal lung samples to determine DNA methylation of all CpGs in a 256bp region displayed as CpG units. Red lines indicate those CpG units containing the CpGs which were originally identified as differentially methylated in EPIC and TCGA. Two tailed t-test p-values. **D:** *NHLRC1* mRNA expression analysis by real time PCR in 92 samples of the lung tumor versus adjacent normal sample set. Whiskers indicate 5-95 percentile. Two tailed t-test p-values, ****: p<0.0001

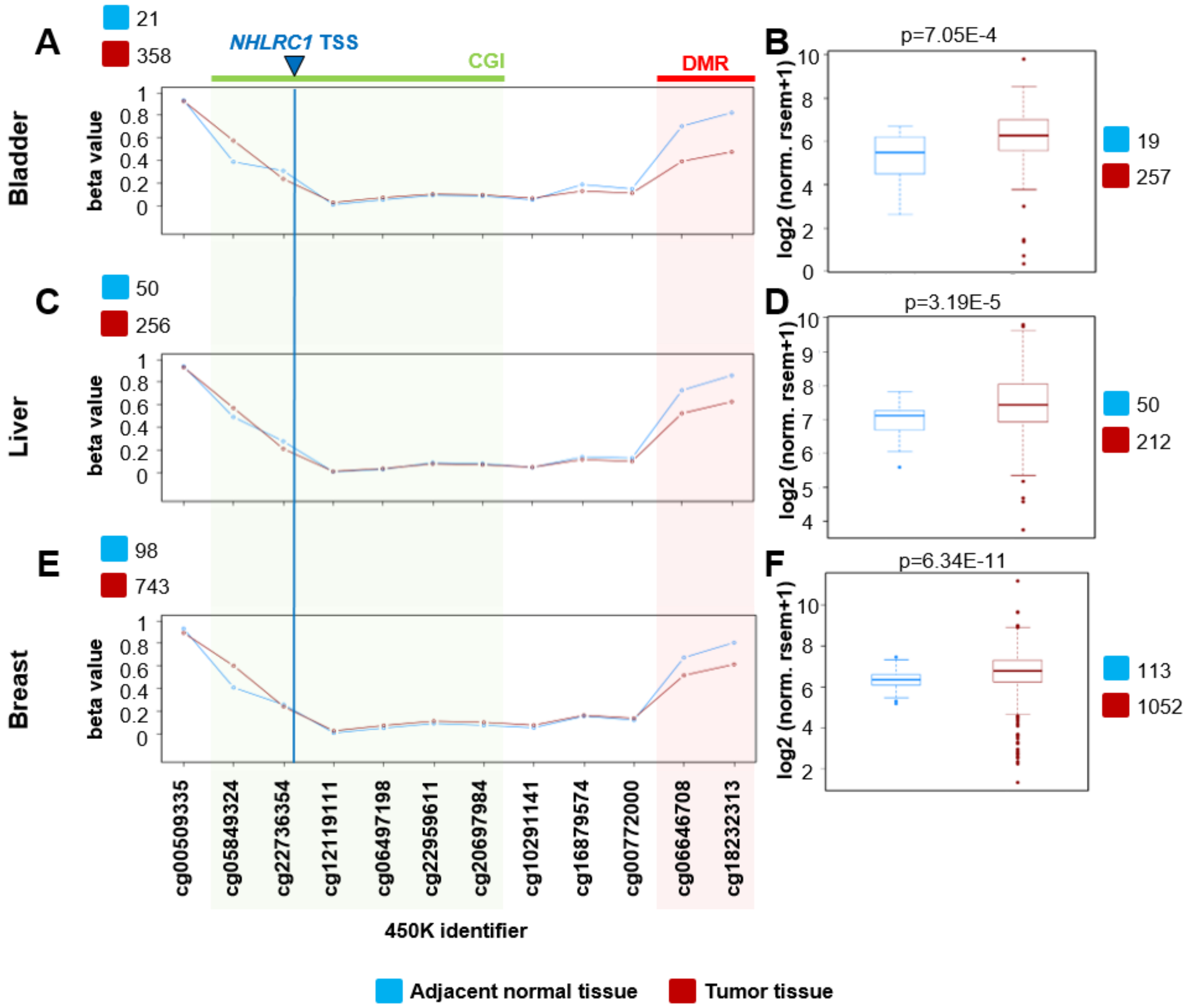

Figure S3. TCGA 450K DNA Methylation and RNA-seq Data in Tumor versus Adjacent Normal Tissues other than Lung for *NHLRC1*. A, C, E: Mean DNA Methylation from 450K data. **B, D, F:** *NHLRC1* gene expression from Illumina HiSeq RNA-seq data. RNA-seq p-values were calculated by Wilcoxon rank sum test. Tumor tissue: red, adjacent normal tissue: blue. Red shading depicts two of the three candidate CpGs. Green shading indicates probes within the CpG island (CGI). Blue arrows refers to the position of *NHLRC1* transcription start site (TSS). TCGA studies: Bladder: bladder urothelial carcinoma **(A, B)**, Liver: liver hepatocellular carcinoma **(C, D)**, Breast: breast invasive carcinoma **(E, F)**, norm.: normalized, rsem: RNA-Seq by Expectation Maximization. Images were produced using TCGA Wanderer platform and modified.

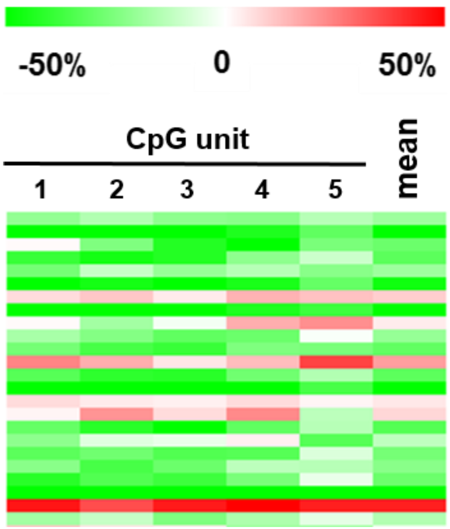

Figure S4. Heatmap of Lung Tumor versus Adjacent Normal Lung Paired Samples of Replication Set II analysed with MassARRAY. Color scheme indicates 50% hypomethylation to 50% hypermethylation in pairs of tumor versus adjacent normal samples.

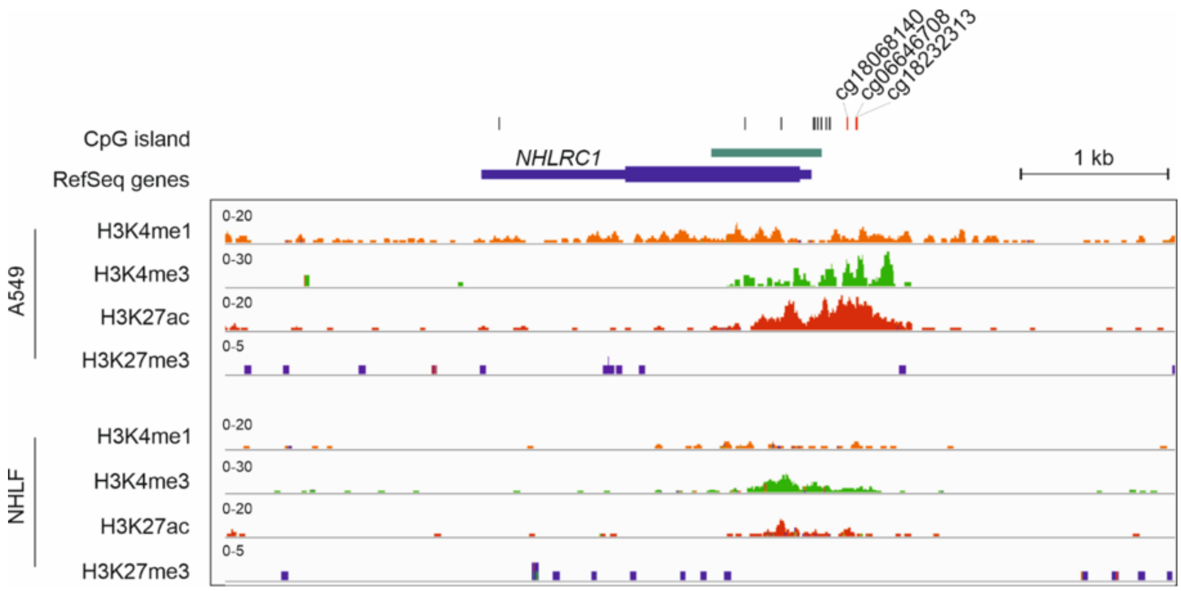

Figure S5. Relative Position of *NHLRC1* to cg06646708 and the ENCODE Histone Marks. ENCODE ChIP-seq peaks are shown for H3K4me3 indicating active promoters and for H3K4me1 and H3K27ac which indicate active enhancers. ENCODE ChIP-seq peak tracks for A549 lung cancer cell line and the lung fibroblast cell line NHLF are displayed. Red ticks indicate the position of the three differentially methylated 450K probes in tumor versus normal tissue.

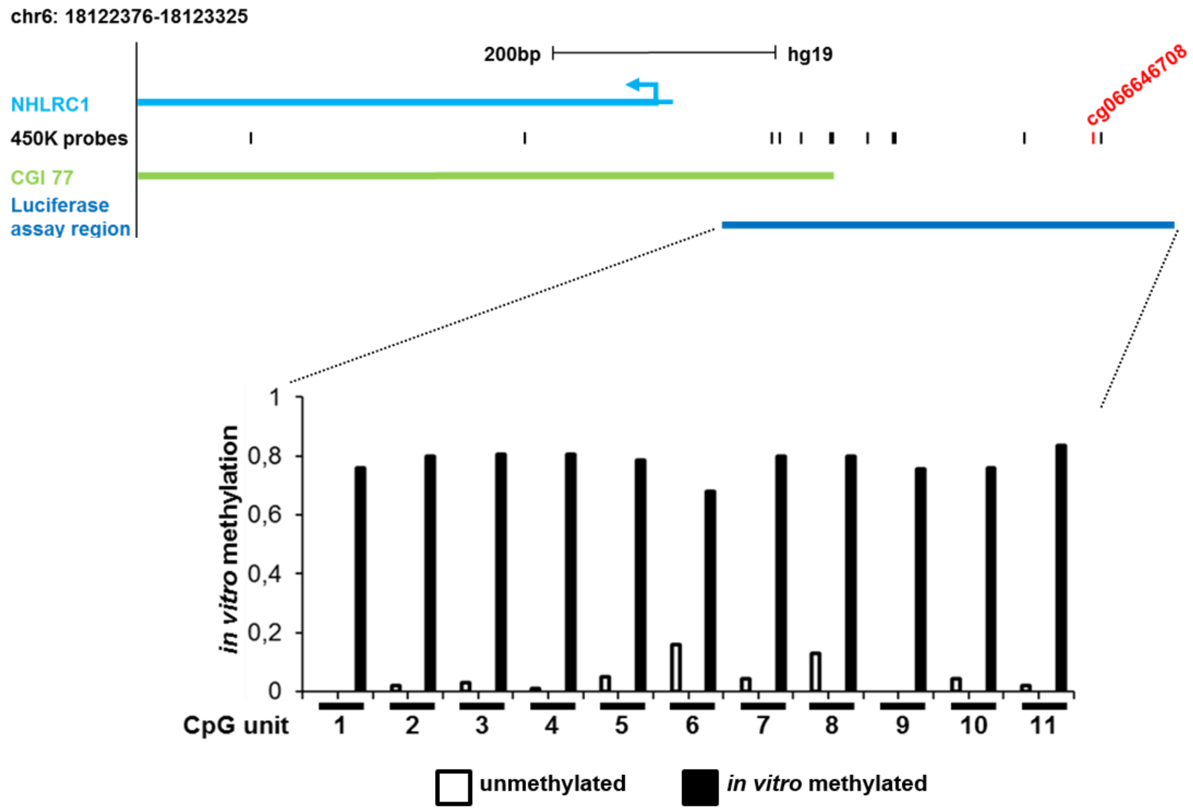

Figure S6. Relative Position of *NHLRC1* to the *in vitro* methylated Luciferase assay DMR. The efficacy of *in vitro* methylation with *MSssI* CpG methyltransferase was tested by MassARRAY.

3. Supplementary Raw Blots

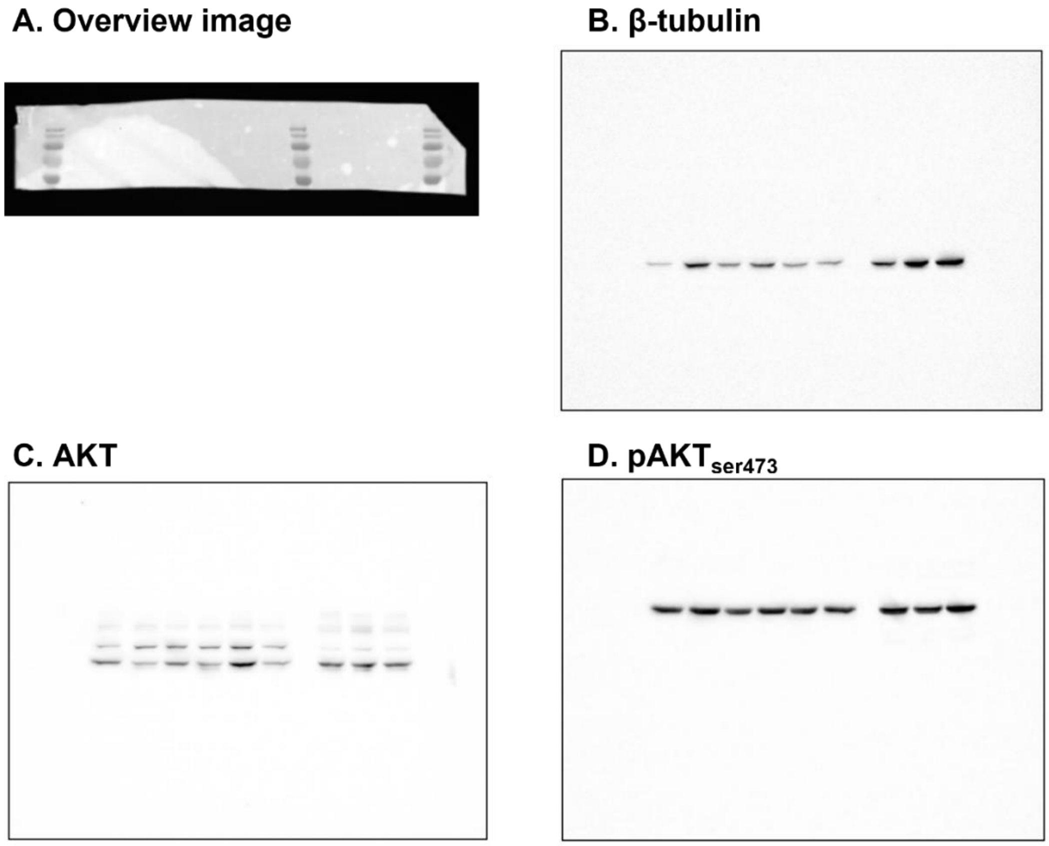

Raw Blot S1. Raw blots of siRNA knockdown of *NHLRC1* in A549. A. Overview image. **B.** Incubation with β-tubulin antibody. **C.** Incubation with AKT antibody. **D.** Incubation with pAKTser473 antibody

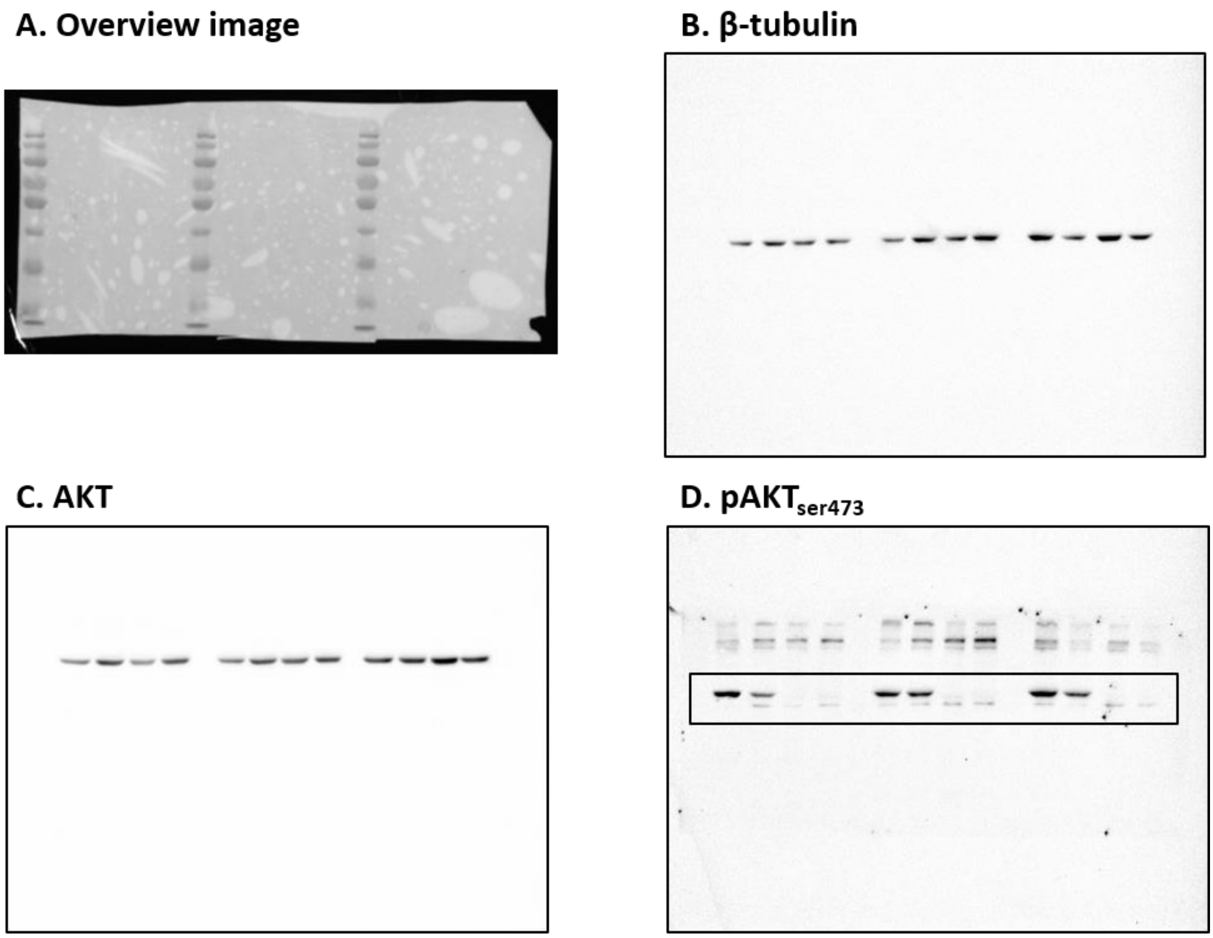

Raw Blot S2. Raw blots of siRNA knockdown of *NHLRC1* in H1299. A. Overview image. **B.** Incubation with β-tubulin antibody. **C.** Incubation with AKT antibody. **D.** Incubation with pAKTser473 antibody

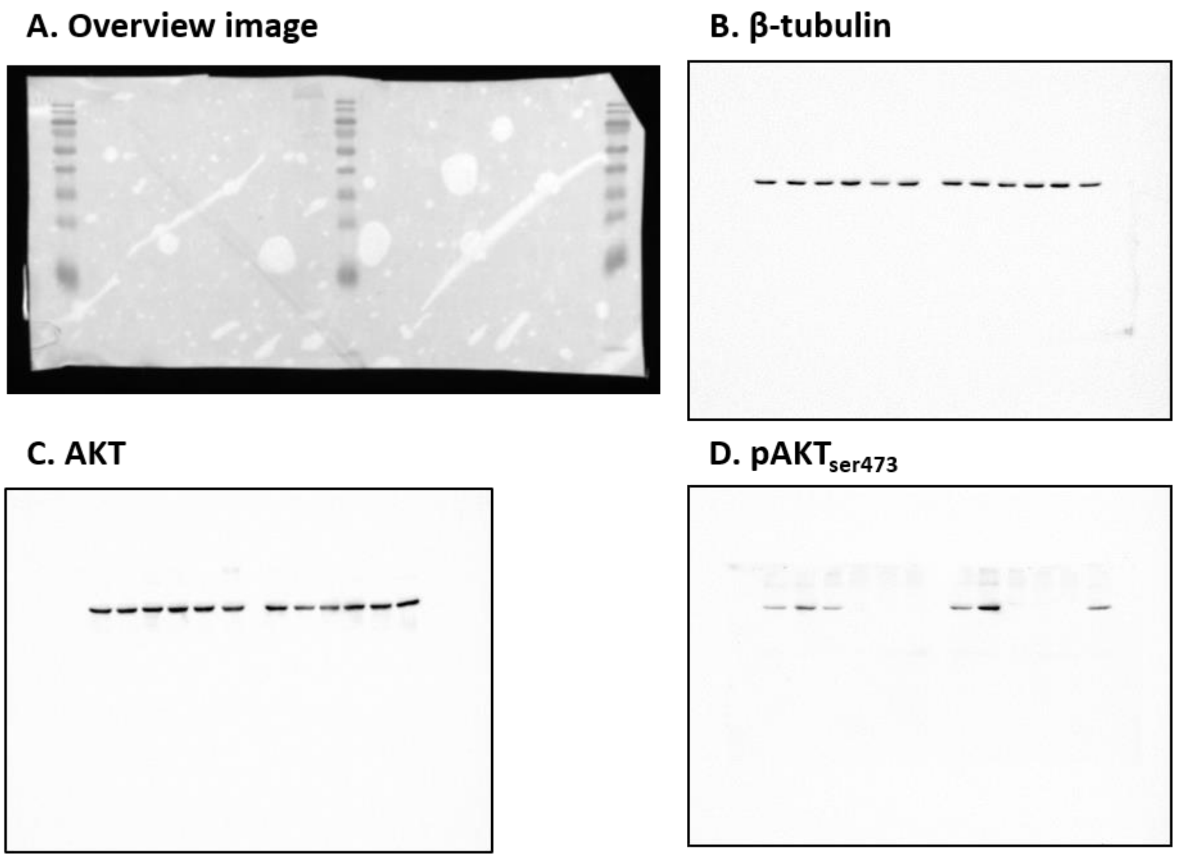

Raw Blot S3. Raw blots of *NHLRC1* overexpression in H1299. A. Overview image. **B.** Incubation with β-tubulin antibody. **C.** Incubation with AKT antibody. **D.** Incubation with pAKTser473 antibody

4. Supplementary R-Script

```
########################################################################################################################
##
## 01_loading_all.R
##
## creator: Yassen Assenov and Christian Faltus
## ------------------------------------------------------------------------------------------------------------------------
## Loads, filters and normalizes all blood samples (EPIC + Kere datasets).
########################################################################################################################
##

DRIVE.PROJECTS <- "Z:"
setwd(file.path(DRIVE.PROJECTS, "USER/EPIC/450K discovery/scripts"))

## L I B R A R I E S ##########################################################################################

suppressPackageStartupMessages(library(doParallel))
suppressWarnings(suppressPackageStartupMessages(library(RnBeads)))
theme_set(theme_bw())

## M A I N
##############################################################################################################

## Adjust analysis options
rnb.options(
```

```
analysis.name = "EPIC_analysis",
        identifiers.column = "Sample_Name",
        region.types = c("genes", "promoters", "cpgislands"),
        normalization.method = "bmiq",
        normalization.background.method = "none",
        filtering.missing.value.quantile = 0,
        inference = TRUE,
        inference.reference.methylome.column = "Type",
        exploratory = FALSE,
        differential = FALSE,
        export.to.bed = FALSE,
        export.to.trackhub = character(),
        disk.dump.big.matrices = FALSE)

## Register parallel processing
#logger.start(fname = NA)
#parallel.setup(2L)
#logger.close()

## ---------------------------------------------------------------------------------------------------------------------
## Load the sample annotation table

sample.annotation <- read.csv("../idat/Vineis_Severi/sample_annotation.csv", check.names = FALSE, stringsAsFactors = FALSE)
#colnames(sample.annotation)[colnames(sample.annotation) == "Sample_Name"] <- "ID"

## Remove whole blood and PBMC samples
#sample.annotation <- sample.annotation[-which(sample.annotation[, "Type"] %in% c("Whole blood", "PBMC")), ]

## Set appropriate types for the traits
sample.annotation[, "Status"] <- factor(sample.annotation[, "Status"])
levels(sample.annotation[, "Status"]) <- tolower(levels(sample.annotation[, "Status"]))
sample.annotation[, "Type"] <- factor(sample.annotation[, "Type"])
sample.annotation[, "smoking_status"] <- factor(sample.annotation[, "smoking_status"])
sentrix.ids <- sample.annotation[, "Sentrix_ID"]
sample.annotation[, "Sentrix_ID"] <- factor(sentrix.ids, levels = as.character(unique(sort(sentrix.ids))))
sample.annotation[, "Sentrix_Position"] <- factor(sample.annotation[, "Sentrix_Position"],
        levels = paste0("R0", rep(1:6, each = 2), "C0", 1:2))
rm(sentrix.ids)

## ---------------------------------------------------------------------------------------------------------------------
## Run RnBeads

dir.idat <- "../idat/Vineis_Severi" # in this example, we also keep the sample annotation table in this directory
dir.reports <- "../Loading" # this directory must not exist

## Run loading, filtering, normalization and annotation inference
rnb.set <- rnb.run.analysis(dir.reports = dir.reports, data.source = list(dir.idat, sample.annotation))

## Save the filtered dataset
save(rnb.set, file = "../data/rnb.set.RData", compression_level = 9L)

## to extract meth table
meth.table <- meth(rnb.set, row.names = T)
## add samples annotation
sample.anotation <- pheno(rnb.set)
write.table(meth.table, "../ Loading/meth.table.txt", sep="\ t", quote = FALSE)
write.csv(meth.table, "../ Loading/meth.table.csv", quote = FALSE)

########################################################################################################################
##
## 02_model_data.R
##
## creator: Yassen Assenov, Christian Faltus
## ---------------------------------------------------------------------------------------------------------------------
## Extracts all tables with values needed to train prediction models on the EPIC dataset.
```

```
#####################################################################################################################
##

setwd("Z:/USER/EPIC/450K discovery/scripts")

## L I B R A R I E S ##################################################################################################

suppressWarnings(suppressPackageStartupMessages(library(RnBeads)))

## G L O B A L S
####################################################################################################

FILE.DATASET <- "../data/rnb.set_Vineis_Severi.RData"

## M A I N
##########################################################################################################

## ---------------------------------------------------------------------------------------------------------------------
## Load the available dataset, containing sample annotation, methylation values and cell type composition estimates

load(FILE.DATASET) # -> rnb.set
rnb.options(identifiers.column = "Sample_Name")
rm(FILE.DATASET)

## ---------------------------------------------------------------------------------------------------------------------
## Extract the tables needed to build prediction models

## Extract the sample pairing information
sample.ids <- samples(rnb.set)
annotation.values <- pheno(rnb.set)[, c("Pair", "Status", "age", "Predicted Gender", "smoking_status")]
colnames(annotation.values) <- c("Pair", "Status", "age", "gender", "smoking_status")
annotation.values[, "smoking_status"] <- factor(as.character(annotation.values[, "smoking_status"]), levels = c("ex", "current"))

## Extract the methylation values
methylation.values <- t(meth(rnb.set, row.names = TRUE))

## Extract the cell type composition estimates
cell.type.values <- rnb.set@inferred.covariates$cell.types
rownames(cell.type.values) <- sample.ids
rm(rnb.set, sample.ids)

## ---------------------------------------------------------------------------------------------------------------------
## Delete samples that will not be used in the prediction models

## Delete pair 13
#i <- which(annotation.values[, "Pair"] == 13)
#if (length(i) != 0) {
#		annotation.values <- annotation.values[-i, ]
#		methylation.values <- methylation.values[-i, ]
#		cell.type.values <- cell.type.values[-i, ]
#}

## Delete the Kere samples
i <- which(annotation.values[, "Status"] == "reference")
if (length(i) != 0) {
		annotation.values <- annotation.values[-i, ]
		methylation.values <- methylation.values[-i, ]
		cell.type.values <- cell.type.values[-i, ]
}
rm(i)

## Delete the currentsmokers samples
#i <- which(annotation.values[, "smoking_status"] == "current")
#if (length(i) != 0) {
# annotation.values <- annotation.values[-i, ]
# methylation.values <- methylation.values[-i, ]
```

```
#  cell.type.values <- cell.type.values[-i, ]
#}
#rm(i)
## ------------------------------------------------------------------------------------------------------------------------
## Save the tables to a file

save(annotation.values, methylation.values, cell.type.values, file = "../data/model.tables.RData",
                compression_level = 9L)
#######################################################################################################################
##
## 03_model_training.R
##
## creator: Christian Faltus, Yassen Assenov
## ------------------------------------------------------------------------------------------------------------------------
## Trains logistic regression models based on the extracted data tables.
#######################################################################################################################
##

setwd("Z:/USER/EPIC/450K discovery/scripts")

## G L O B A L S
#####################################################################################################


## Input file containing the sample annotation, methylation values and cell type composition data
FILE.TABLES <- "../data/model.tables.RData"

## Directory to contain the generated plots and exported tables of biomarker candidates
DIR.RESULTS <- "../PCs_BH_0.05-ALL"

## Threshold to consider a (corrected) p-value significant
PVALUE.THRESHOLD <- 0.05

## M A I N
##############################################################################################################

## ------------------------------------------------------------------------------------------------------------------------
## Load the available data

load(FILE.TABLES) # -> annotation.values, methylation.values, cell.type.values
rm(FILE.TABLES)

## ------------------------------------------------------------------------------------------------------------------------
## Calculate methylation increase (intra-pair methylation difference)

## Construct a mapping from pair ID to the sample indices
pair.to.sample.indices <- tapply(1:nrow(annotation.values), annotation.values[, "Pair"], function(i) {
  if (length(i) != 2) {
    stop("unexpected number of samples in a pair")
  }
  pair.status <- as.character(annotation.values[i, "Status"])
  if (identical(pair.status, c("case", "control"))) {
    return(i)
  } else if (identical(pair.status, c("control", "case"))) {
    return(rev(i))
  }
  stop("unexpected sample status value in a pair")
}
)
pair.to.sample.indices <- sapply(pair.to.sample.indices, identity)
rownames(pair.to.sample.indices) <- c("case", "control")

## Compute methylation differences
methylation.differences <- t(apply(pair.to.sample.indices, 2, function(i) {
  methylation.values[i[1], ] - methylation.values[i[2], ]
}
```

```
))

## -----------------------------------------------------------------------------------------------------------------------
## Principal component analysis of the cell type compositions

pca.result <- prcomp(cell.type.values)
pr.coordinates <- pca.result$x
pr.differences <- apply(pair.to.sample.indices, 2, function(i) {
  pr.coordinates[i[1], 1:2] - pr.coordinates[i[2], 1:2]
}
)

## Create the output directory if necessary
if (!file.exists(DIR.RESULTS)) {
  dir.create(DIR.RESULTS, recursive = TRUE)
}

## Plot PCA coordinates
pdf(file.path(DIR.RESULTS, "PCA_cell_types1vs2.pdf"), width = 7.2, height = 7.2)
point.colors <- c("red", "blue")[as.integer(annotation.values$Status)]
plot(pr.coordinates[, 1], pr.coordinates[, 2], xlab = "Principal component 1", ylab = "Principal component 2", type = "n")
text(pr.coordinates[, 1], pr.coordinates[, 2], labels = as.character(annotation.values$Pair), col = point.colors)
#pdf(file.path(DIR.RESULTS, "PCA_cell_types1vs3.pdf"), width = 7.2, height = 7.2)
#point.colors <- c("red", "blue")[as.integer(annotation.values$Status)]
#plot(pr.coordinates[, 1], pr.coordinates[, 3], xlab = "Principal component 1", ylab = "Principal component 3", type = "n")
#text(pr.coordinates[, 1], pr.coordinates[, 3], labels = as.character(annotation.values$Pair), col = point.colors)
dev.off()
rm(pca.result, point.colors)

## -----------------------------------------------------------------------------------------------------------------------
## Train one linear model for every CpG

pvalues <- apply(methylation.differences, 2, function(meth.differences) {
  cpg.data <- data.frame(
    y = meth.differences,
    c1 = pr.differences[1, ],
    c2 = pr.differences[2, ])
  model.fit <- glm(y ~ c1 + c2, data = cpg.data)
  ## Get p-value for intercept != 0
  summary(model.fit)$coefficients["(Intercept)", "Pr(>|t|)"]
}
)

## Plot a histogram of all p-values
pdf(file.path(DIR.RESULTS, "Histogram_pvalues_uncorrected.pdf"), width = 7.2, height = 6.2)
hist(pvalues, breaks = seq(0, 1, length.out = 101), main = NA, xlab = "P-value", ylab = "Frequency", col = "#000040")
dev.off()

## Correct p-values for multiple testing
pvalues.corrected <- p.adjust(pvalues, method = "BH")

## Plot a histogram of corrected p-values
pdf(file.path(DIR.RESULTS, "Histogram_pvalues_corrected.pdf"), width = 7.2, height = 6.2)
hist(pvalues.corrected, breaks = seq(0, 1, length.out = 101), main = NA, xlab = "P-value", ylab = "Frequency", col = "#000040")
dev.off()

## Plot a Q-Q plot of corrected p-values
pdf(file.path(DIR.RESULTS, "QQ_pvalues_corrected.pdf"), width = 7.2, height = 7.2)
qqplot(pvalues.corrected, seq(0, 1, length.out = length(pvalues.corrected)), xlab = "Observed p-value quantile",
       ylab = "Expected p-value quantile (uniform)", xlim = c(0, 1), ylim = c(0, 1), pch = 16, col = "navy")
dev.off()

## Export tables of differentially methylated sites with their adjusted p-values - CF
pvalues.corrected <- sort(pvalues.corrected[pvalues.corrected < PVALUE.THRESHOLD])
pvalues.corrected <- data.frame("ID" = names(pvalues.corrected),
                               "P-value" = unname(pvalues[names(pvalues.corrected)]), "P-value (corrected)" = unname(pvalues.corrected),
```

```
                    check.names = FALSE, stringsAsFactors = FALSE)
tbl <- cbind(pvalues.corrected, t(methylation.values[, pvalues.corrected[, "ID"]]))
write.csv(tbl, file.path(DIR.RESULTS, "Candidates_methylation_BH0.05.csv"), row.names = FALSE)
tbl <- cbind(pvalues.corrected, t(methylation.differences[, pvalues.corrected[, "ID"]]))
write.csv(tbl, file.path(DIR.RESULTS, "Candidates_increase_all_BH0.05.csv"), row.names = FALSE)
```
